# Supplementary material for: Neutrophils in the Spotlight—An Analysis of Neutrophil Function and Phenotype in ARDS
Source: Int J Mol Sci. 2024 Nov 22;25(23):12547. doi: 10.3390/ijms252312547 (PMC11641705; doi:10.3390/ijms252312547)
Supplement: Supplementary file 1 [file ijms-25-12547-s001.zip › ijms-3297322-supplementary.pdf]

Table S 1: Detailed information about included patients

	**Underlying disease**	**ARDS**	**Reason for ARDS**	**Trigger** pulmonary = 0 systemic = 1	**VV-ECMO**	**VA-ECMO**	**SMOKER**	**Ventilation** Tube = 0 Tracheal-Cannula =1	**Macroscopic** Mucous = 1 Sanguinous = 2 Serous(clear) = 3	**Detected Pathogens**	**DIALYSIS**
1	liver abscess	1		1	0	0	1	0	1		0
2	liver abscess	1		1	0	0	1	0	1		0
3	Pneumococcus-Pneumonia	1	Infection	0	1	0	1	0	2	Streptococcus Pneumonia	1
4	Pneumococcus-Pneumonia	1	Infection	0	1	0	1	0	2	Streptococcus Pneumonia	1
5	Aortic valve stenosis:Transcatheter aortic-valve implantation (TAVI)	0			0	0	0		3		0
6	Bleeding of the upper nasopharynx in a patient with known Osler's disease (blood aspiration)	1	Aspiration	1	0	0	0	0	2		0
7	pneumococcal pneumonia	1	Infection	1	1	0	1	0	2	pneumococci	1
8	Septic shock due to oral abscess	1	Infection	0	0	0	1	0	1	Neisseria macacae, Staph. aureus, greening streptococci	
9	Locally infiltrating the thyroid cartilage and lymphogenous metastasizing	0			0	0	1	1	2		

10	Aortic valve stenosis:Transcatheter aortic-valve implantation (TAVI)	0			0	0	1	0	3		0
11	Septic shock with right neck phlegmon	1	Systemic inflammation due to sepsis	0	0	0	0	1			1
12	Acute necrotizing anal fissure and perianal abscess	1	Systemic inflammation due to sepsis/pneumonia	1	0	0	0	0	2	E. coli, Pseudomonas aeruginosa, Morganella Morganii	0
13	Septic shock in secondary peritonitis after anastomotic insufficiency of a small bowel anastomosis	0			0	0	0	0	1		0
14	Respiratory Insufficiency in Pneumonia	1	infection	1	0	0		0	2	Klebsiella, Staphylococcus epidermidis	0
15	aortic valve stenosis: AKE	0			0	0	1	0	3		0
16	Post-dissection aneurysm: Sonographically guided puncture of the right common femoral artery	0			0	0	0	0	3		0
17	Lung metastasis lower lobe right: re-thoracotomy right	0			0	0	0	0	3		0
18	Laparotomy for enterocutaneous fistula in high-output incisional hernia in Zn sigmoid carcinoma	0			0	0	0	0	3		0

19	MI in endocarditis: partial resection, annuloplasty Physioring	0	0	0	0	0	2	0
20	Heart pacemaker implantation	0	0	0	0	0	3	0
21	Aortic valve regurgitation: AKE	0	0	1	0	0	3	0
22	Torsade de Point tachycardia / Zn VVI-ICD b. Zn ventricular fibrillation: DDD implantation	0	0	0	0	0	3	0
