# Supplementary material for: Neutrophils in the Spotlight—An Analysis of Neutrophil Function and Phenotype in ARDS
Source: Int J Mol Sci. 2024 Nov 22;25(23):12547. doi: 10.3390/ijms252312547 (PMC11641705; doi:10.3390/ijms252312547)
Supplement: Supplementary file 1 [file ijms-25-12547-s001.zip › ijms-3297322-supplementary_V3.pdf]

Supplementary results of the experiments

Table S2. Overview of the medians, interquartile ranges (IQR) and the number of experiments (n) testing the percentages of non-living PMN

Percentage of non-living PMN	Median	IQR	n
cPMN without ARDS	0.2	0.2	36
cPMN with ARDS	0.4	0.5	29
tPMN without ARDS	36	32	32
tPMN with ARDS	20	38	30

Table S3. Overview of medians, interquartile ranges IQR, the number of trials n and the significance p of MFI(CD11b).

MFI(CD11b)	Median	IQR	n
cPMN without ARDS	337	288	12
cPMN with ARDS	416	633	10
tPMN without ARDS	332	1298	12
tPMN with ARDS	666	1022	9

Table S4. Overview of mean values (MV), standard deviation (SD), number of trials (n) and significance p of MFI(CD66b)

AFU (CD66b)	MV	SD	n
cPMN without ARDS	110	25	12
cPMN with ARDS	194	69	10
tPMN without ARDS	424	305	12
tPMN with ARDS	505	329	9

Table S5. Overview of mean values (MV), standard deviation (SD), number of trials (n) and significance p of MFI(CD62L)

AFU (CD62L)	MV	SD	n
cPMN without ARDS	170.3	19.6	11
cPMN with ARDS	72.6	14.9	8
tPMN without ARDS	44.6	26.0	12
tPMN with ARDS	44.7	26.1	10

Table S6. Overview of medians, interquartile ranges (IQR) and number of trials (n) of MFI(LOX-1).

AFU(LOX-1)	Median	IQR	n
cPMN without ARDS	7.9	3.0	11
cPMN with ARDS	9.7	9.3	9
tPMN without ARDS	203.4	835.4	12
tPMN with ARDS	235.0	561.3	9

Table S7. Overview of mean values (MV), standard deviation (SD), number of trials (n) and significance p of MFI(CD49d)

AFU (CD49d)	MV	SD	n
cPMN without ARDS	15.2	8.8	12
cPMN with ARDS	13.8	7.1	8
tPMN without ARDS	54.8	30.0	12
tPMN with ARDS	12.6	7.4	7

Table S8. Overview of mean values (MV), standard deviation (SD), number of trials (n) and significance p of MFI(CD29).

AFU (CD29)	MV	SD	n
cPMN without ARDS	15.0	4.9	12
cPMN with ARDS	14.5	10.4	9
tPMN without ARDS	53.2	42.3	11
tPMN with ARDS	20.2	15.1	9

Table S9. Overview of medians, interquartile ranges (IQR) and number of trials (n) of MFI(fMLP-R).

MFI (fMLP-R)	Median	IQR	n
cPMN without ARDS	26,4	22,8	13
cPMN with ARDS	34,4	19,7	9
tPMN without ARDS	389,1	1442,7	11
tPMN with ARDS	448,0	1591,9	9

Table S10. Overview of medians, interquartile ranges (IQR) and number of trials (n) of blank value MFI(DHR).

Blank value MFI(DHR)	Median	IQR	n
cPMN without ARDS	3,2	2,6	12
cPMN with ARDS	3,5	1,5	9
tPMN without ARDS	15,8	59,6	11
tPMN with ARDS	14,6	35,9	9

Table S11. Overview of medians, interquartile ranges (IQR) and number of trials (n) of blank value MFI(DHR) -fMLP+TNF α

MFI(DHR)-FMLP + TNFα	Median	IQR	n
cPMN without ARDS	42	16	12
cPMN with ARDS	30	36	10
tPMN without ARDS	15	31	11
tPMN with ARDS	15	17	9

Table S12. Overview of medians, interquartile ranges (IQR) and number of trials (n) of blank value MFI(DHR)-PMA.

MFI(DHR)-PMA	Median	IQR	n
cPMN without ARDS	410	1064	13
cPMN with ARDS	403	547	9
tPMN without ARDS	138	303	12
tPMN with ARDS	45	146	10

Supplement

Measurement details of the flow cytometry

Table S13. Fluorescent dyes of the antibodies used, their channels and laser wavelengths.

Antibody	Coupled dye	Fluorescence channel (H = Height)	Absorption	Emission
CD11b	PE	FL2-H	488 nm	575 nm
CD62L	FITC	FL1-H	488 nm	525 nm
CD66b	APC	FL4-H	633 nm	660 nm
LOX-1	APC	FL4-H	633 nm	660 nm
CD49d	PE	FL2-H	488 nm	575 nm
CD29	FITC	FL1-H	488 nm	525 nm
FMLP-Receptor	APC	FL4-H	633 nm	660 nm

Table S14. Channels and wavelengths of the lasers of the substances used in the ROS experiment.

ROS experiment	Fluorescence channel (H = Height)	Absorption	Emission
Propidiumiodide	FL3-H	535 nm	617 nm
Rhodamine-123	FL1-H	508 nm	528 nm
